# The complete chloroplast genome sequence of *Carya illinoinensis* cv. wichita and its phylogenetic analysis

**DOI:** 10.1080/23802359.2020.1768925

**Published:** 2020-06-01

**Authors:** Gang Feng, Zheng-Hai Mo, Fang-Ren Peng

**Affiliations:** a Co-Innovation Center for the Sustainable Forestry in Southern China, Nanjing Forestry University, Nanjing, China; b Institute of Botany, Chinese Academy of Sciences, Nanjing, Jiangsu Province, China

**Keywords:** *Carya illinoinensis*, chloroplast genome, phylogeny

## Abstract

*Carya illinoinensis* is an important nut tree with high economic and ecological values. Here, we presented the complete chloroplast (cp) genome sequence of *C. illinoinensis* cv. wichita. The whole cp genome is 160,532 bp in length, displaying a typical quadripartite structure with a large single-copy (LSC) of 897,99 bp, a small single-copy (SSC) region of 18,751 bp, and a pair of inverted repeats (IRs) of 25,991 bp. A total of 128 genes were predicted to contain in the whole cp genome, including 83 protein-coding genes, 37 tRNA genes, and 8 rRNA genes. The GC contents of the cp genome is 36.19%. Phylogenomic analysis suggested *Carya illinoinensis* as a sister species of *C. cathayensis*, *C. kweichowensis*, and *Annamocarya sinensis*.

*Carya illinoinensis*, also known as pecan, is native to North America with a broad natural distribution, which extends from Mexico to United States (Mo et al. 2018). Nowadays, it has been commercially cultivated in many countries including Australia, South Africa, China, Brazil, and Peru (Casales et al. 2018). *Carya illinoinensis* is an economically important member of *Carya* genus in Juglandaceae family. Previously, the classification of *C. illinoinensis* was determined mainly based on its morphology and a systematic categorization on the basis of genomic data is still lacking. As the complete cp genome is helpful for phylogenetic analysis (Zhao et al. 2019; Yang et al. 2020), in our study, the cp genome of *C. illinoinensis* was *de novo* assembled and compared with other cp genomes so as to reveal its phylogeny position on the molecular level.

Fresh leaves of *C. illinoinensis* cv. wichita were collected from the experimental farm of Nanjing Forestry University (Nanjing, China, 119°9′10.64″E, 31°52′44.76″N) and were deposited at Nanjing Forestry University (No. NFUFG001). Genomic DNA was extracted using the DNeasy Plant Mini Kit (Qiagen, Valencia, CA, USA). The DNA was stored at -80 °C in our lab until further analyzed. A paired end library with an approximate insert size of 350 bp was built and sequenced on the Illumina NovaSeq system (Illumina, San Diego, CA, USA). Totally, 4427 Mb of raw data were generated. Raw reads were filtered using the Trimmomatic v0.32 (Bolger et al. 2014). The filtered sequences were *de novo* assembled and annotated by NOVOPlasty (Dierckxsens et al. 2017) and DOGMA (Wyman et al. 2004), respectively. The annotated cp genome was deposited in GenBank under the accession number MT044463.

The cp genome of *C. illinoinensis* cv. wichita was a circular double-stranded DNA of 160,532 bp containing two inverted repeat (IR) regions of 25,991 bp each, separated by a large single-copy (LSC) and a small single-copy (SSC) regions of 89,799 bp and 18,751 bp, respectively. A total of 128 genes were annotated, including 83 protein-coding genes, 37 tRNA genes, and 8 rRNA genes. There were 18 intron-containing genes with 16 contained one intron and 2 contained two introns. The overall GC content of *C. illinoinensis* cp genome is 36.19% and the ratios in LSC, SSC, and IR regions were 33.79, 29.90, and 42.58%, respectively.

The phylogenetic position of *C. illinoinensis* was determined using the maximum-likelihood (ML) method based on 20 complete cp genomes. Sequence alignment was conducted by MAFFT (Katoh and Standley 2013) and phylogenetic tree was constructed by IQ-tree. The result (Figure 1) supported the position of *C. illinoinensis* as a sister species of *C. cathayensis*, *C. kweichowensis*, and *Annamocarya sinensis*.

**Figure 1.**
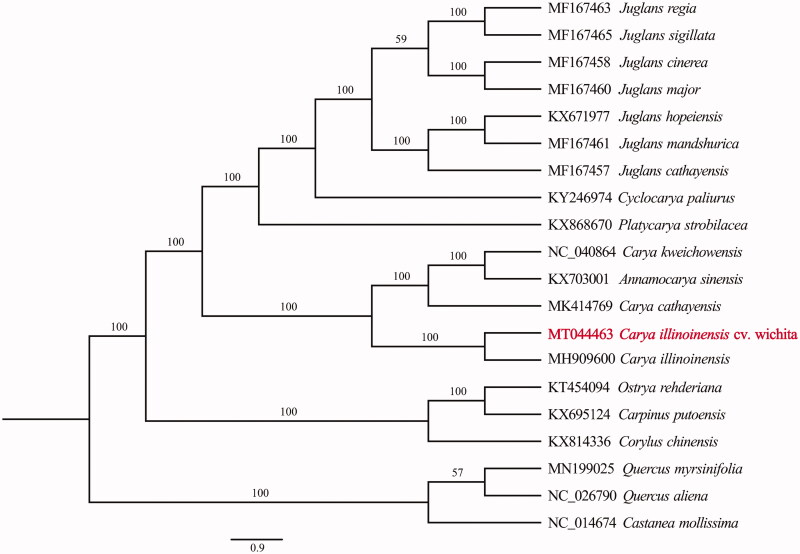
Phylogenetic position of *C. illinoinensis* based on 20 complete cp genomes. Phylogenetic tree was constructed using ML method. The bootstrap support values were shown at the branches.

## Data Availability

The data that support the findings of this study are openly available in NCBI at http://www.ncbi.nlm.nih.gov/, reference number MT044463.
